# Supplementary material for: Variation in personality can substitute for social feedback in coordinated animal movements
Source: Commun Biol. 2021 Apr 13;4:469. doi: 10.1038/s42003-021-01991-9 (PMC8044162; doi:10.1038/s42003-021-01991-9)
Supplement: Supplementary file 2 — Supplementary Information [file 42003_2021_1991_MOESM2_ESM.pdf]

Variation in personality can substitute for social feedback in coordinated animal movements

Planas-Sitjà I[1], Deneubourg J-L[2] & Cronin A L[1]
[1]*Department of Biology, Tokyo Metropolitan University, 1-1 Minami-Osawa, Hachioji, Tokyo, 192-0397, Japan*
[2]*Center of Nonlinear Phenomena and Complex Systems (CENOLI) - CP 231, Université libre de Bruxelles, Campus la Plaine NO.5, Boulevard du Triomphe, 1050 Bruxelles, Belgium*

Supplementary Material:

Our simulations aim to describe how personality and positive feedbacks interact during collective movements. In this file we devote the first section to the results of simulations for each specific scenario and condition. The second section explains additional details of the analytical model and presents some supplementary results.

1. Results of the simulations for each scenario and condition

Figures S1-S6 summarise the results of simulated movements split by condition/scenario. Figure S1 shows Despotic-bimodal results for each different number of leaders (see Figure S2 for the bimodal index); Figure S3 shows Despotic-unimodal results (with Despotic-bimodal results, with a ratio of leaders-followers 5:5, for comparison); Figure S4 shows Democratic-bimodal results for each different number of leaders (see Figure S5 for the bimodal index); Figure S6 shows Democratic-unimodal results (with Democratic-bimodal results, with a ratio of leaders-followers 5:5, for comparison).

Bimodality was more easily achieved with an intermediate number of leaders/followers in the Despotic scenario and Bimodal condition (Fig. S1, S2). In such a scenario, when removing the effect of the feedback between followers and initiator (Bim-constant and Bim-Cα), increasing the number of leaders within group (~7 instead of ~5) improved bimodality. With few leaders, bimodality appears more often with small differences between individuals, although the opposite is true for simulations with many leaders.

In the Despotic scenario, Unimodal condition (Fig. S3), we show that bimodal response patterns are more likely if individuals are more similar. Increasing the degree of differences tends to maximise the number of failed movements (less numbers of followers). In these cases we can expect a distributed leadership situation to arise, in which all individuals are equally social attractive and promote the same probability of following, with no emergence of leaders or followers (Couzin and Krause, 2003; Gérard et al., 2020; King and Cowlishaw, 2009; Leca et al., 2003; Strandburg-Peshkin et al., 2015; Sueur and Petit, 2008).

In the Democratic scenario, bimodality was achieved when considering small range of differences between individuals under both Bimodal (Fig. S4, S5) and Unimodal (Fig. S5) conditions. Feedback between followers and the initiator, and more precisely non-linear feedback, increase bimodality.

Despotic scenario

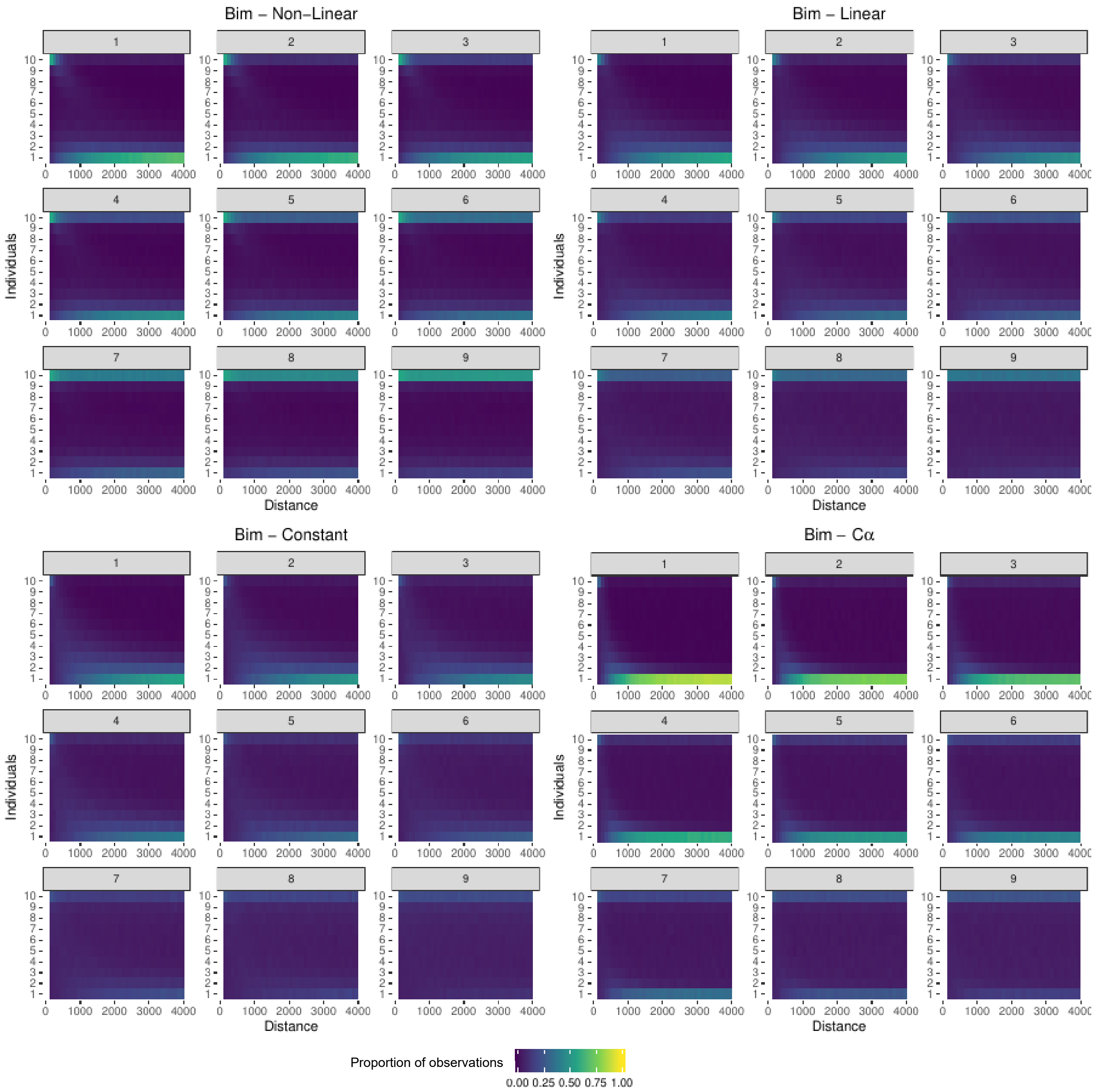

Figure S1. Results of the simulations for the despotic scenario and bimodal condition for different numbers of leaders. Bim-non-linear corresponds to bimodal condition with non-linear cancellation rate; Bim-linear is the bimodal condition with linear cancellation rate; Bim-constant is the bimodal condition with a constant cancellation rate; Bim-Cα is the bimodal conditions with the cancellation rate proportional to α' (from the probability of joining). The y-axis corresponds to the number of individuals following a movement, and the x-axis is the distance between both normal distributions used to create the bimodal distribution of α' (probability of joining a movement). The number in the grey box on the top of each plot indicates the number of leaders (number of individuals taken from the distribution with lower α', individuals with higher probability of joining a movement). The colour is the proportion of observations for a given set of simulations, and thus ranges from 0 to 1 with lighter colours indicating higher values.

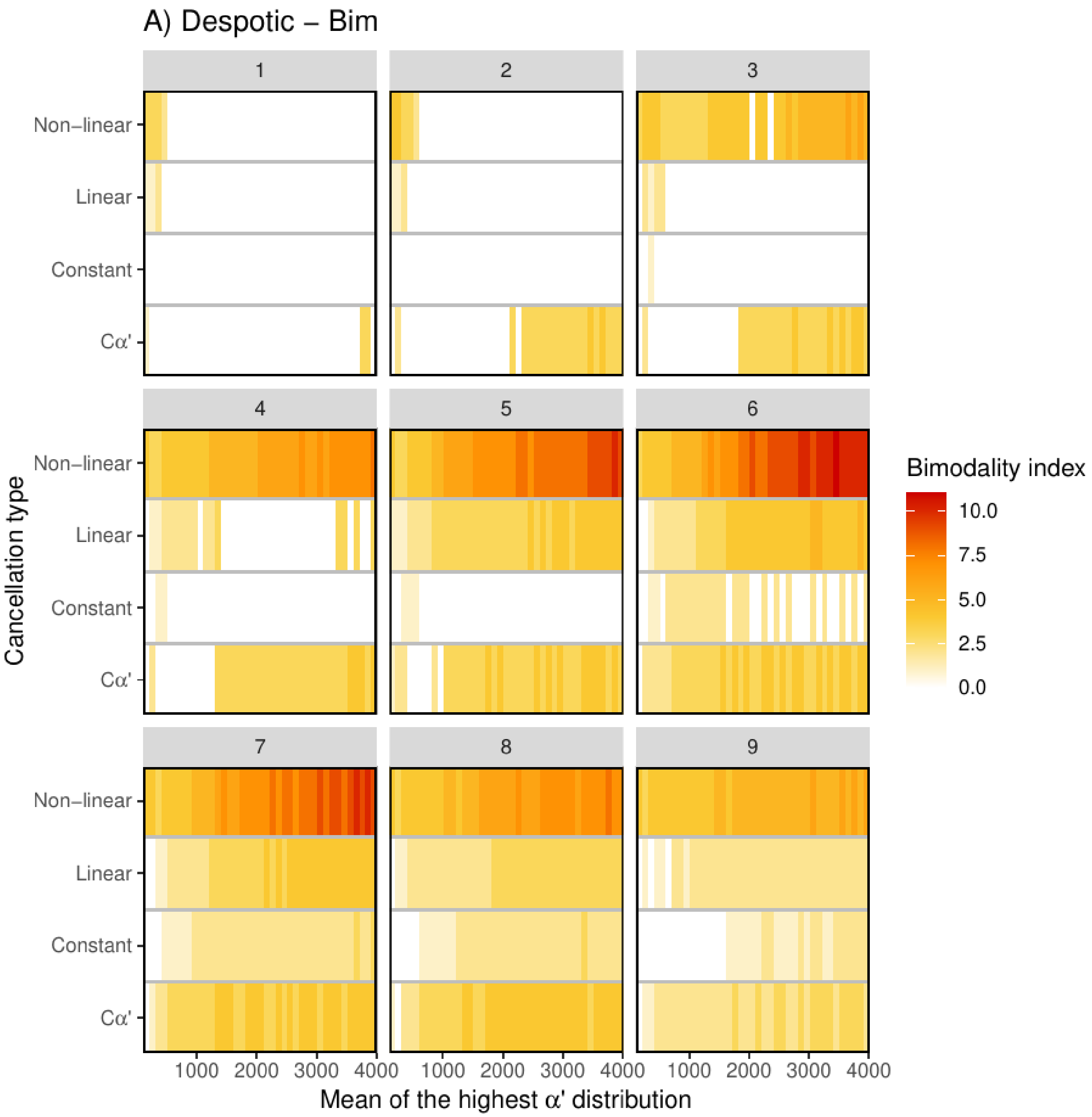

Figure S2. Scenarios where bimodality was produced under Bimodal condition for Despotic scenarios. Y-axis indicates the cancellation type and the x-axis is the range of difference in *α'* values. The number on the top of each figure indicates the number of leaders. The first column of each graph corresponds to the control simulation without personality as *α'* = 160 for all individuals. For the following columns the mean of the low α' distribution was fixed at 160, while the mean of the highest *α'* distribution is shown on the x-axis. The colour scale indicates the bimodality index (see methods for computation details); the higher the index, the higher the degree of bimodality.

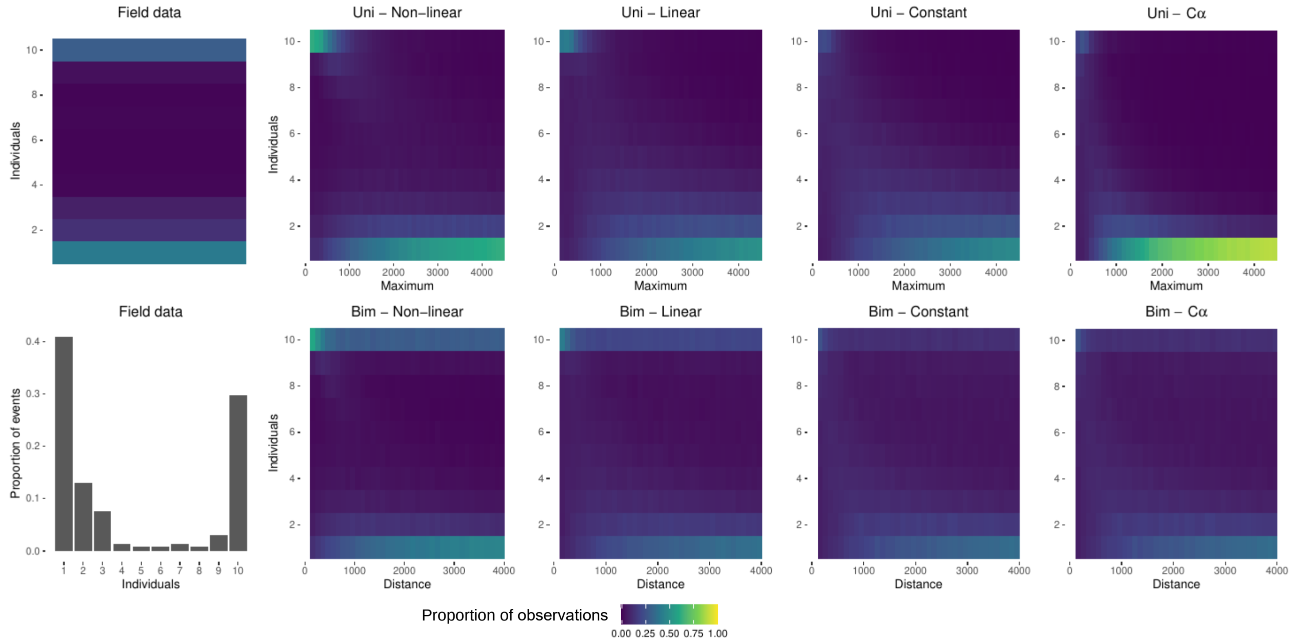

Figure S3. Results of the simulations for the Despotic scenario and Unimodal condition. Uni-non-linear corresponds to Unimodal condition with non-linear cancellation rate; Uni-linear is the Unimodal condition with linear cancellation rate; Uni-constant is the Unimodal condition with a constant cancellation rate; Uni-Cα is the Unimodal condition with constant cancellation rate proportional to *α'* (from the probability of joining). For comparison, we plot the Bimodal condition with a proportion of 5:5 leaders:followers. Bim-non-linear corresponds to Bimodal condition with non-linear cancellation rate; Bim-linear is the Bimodal condition with linear cancellation rate; Bim-constant is the Bimodal condition with a constant cancellation rate; Bim-Cα is the Bimodal conditions with constant cancellation rate proportional to *α'* (from the probability of joining). The y-axis corresponds to the number of individuals following a movement. In the case of Unimodal condition, the x-axis is the maximum possible value of α' for a given simulation. In the case of Bimodal condition, the x-axis is the distance between both normal distributions used to create the bimodal distribution of *α'* (probability of joining a movement). The colour is the proportion of observations for a given set of simulations, and thus ranges from 0 to 1 with lighter colours indicating higher values. On the bottom-left, we show the experimental observation results presented as a two-dimensional representation for comparison with simulation results (which can be considered 'top-views' of the same).

Movie S1:

Legend of Movie S1: Summary of the results of the simulations for the Despotic scenario and Bimodal condition for different numbers of leaders. For Unimodal condition we used a uniform distribution of *α'* instead of a normal distribution. The results obtained with both distributions are qualitatively the same. From left to right, Non-linear corresponds to condition with non-linear cancellation rate; Linear is the condition with linear cancellation rate; Constant is the condition with a constant cancellation rate; Constant-alpha is the conditions with the cancellation rate proportional to α' (probability of joining). The x-axis (N) corresponds to the number of individuals following a movement, the y-axis (Observation) is the proportion of observation of each number of individuals following a movement for a given set of simulations, and the z-axis (Mean) is the distance between both normal distributions used to create the bimodal distribution of α' (probability of joining a movement). N Leaders on the top of each plot indicates the number of leaders (number of individuals taken from the distribution with lower α', individuals with higher probability of joining a movement). The bar colour, like the y-axis, is the proportion of observations for a given set of simulations, and thus ranges from 0 to 1.

Democratic scenario

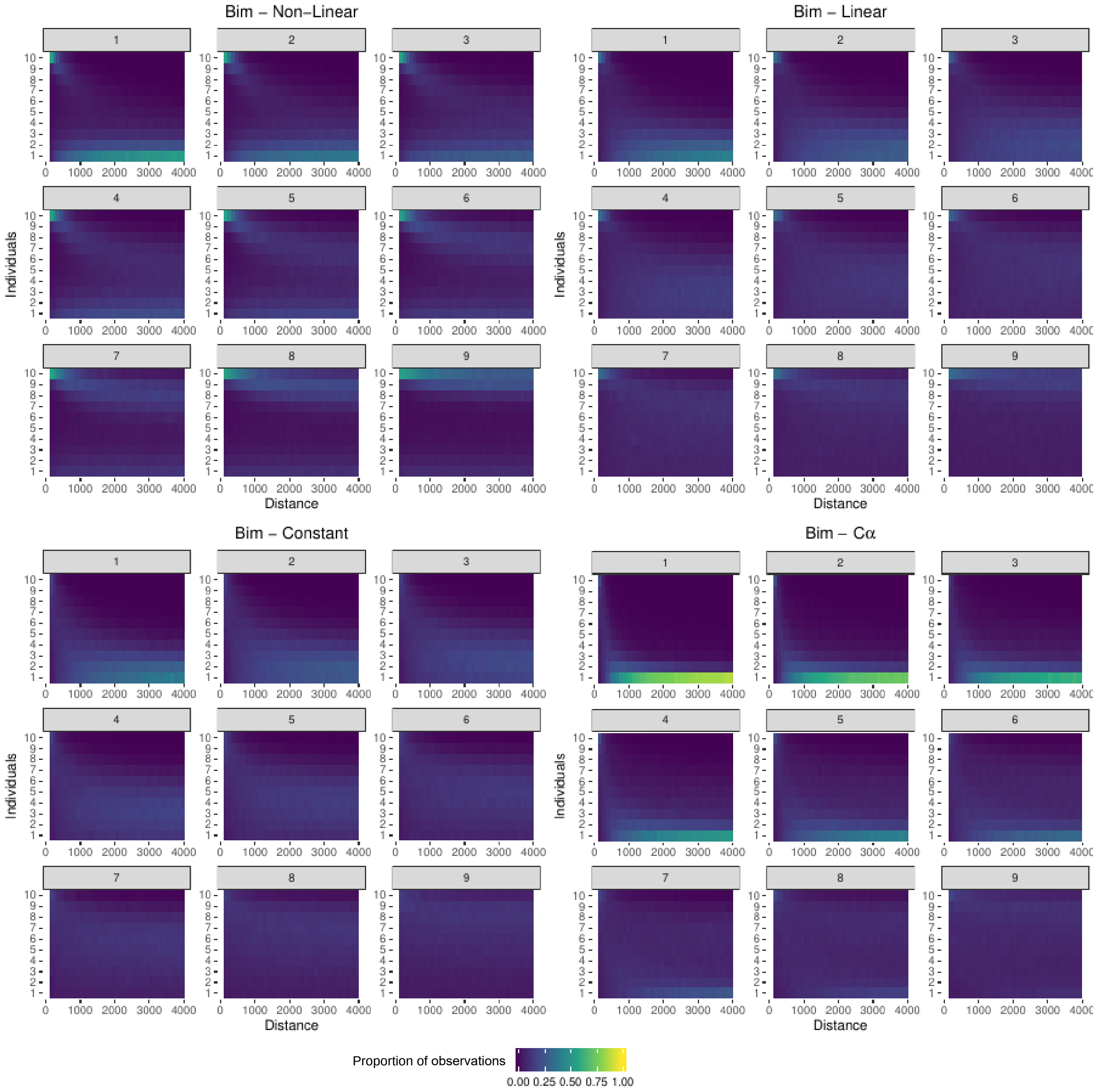

Figure S4. Results of the simulations for the Democratic scenario and Bimodal condition. Bim-non-linear corresponds to Bimodal condition with non-linear cancellation rate; Bim-linear is the Bimodal condition with linear cancellation rate; Bim-constant is the Bimodal condition with a constant cancellation rate; Bim-Cα is the Bimodal conditions with thecancellation rate proportional to *α'* (from the probability of joining). The y-axis corresponds to the number of individuals following a movement, and the x-axis is the distance between both normal distributions used to create the bimodal distribution of *α'* (probability of joining a movement). The number in the grey box on the top of each plot indicates the number of leaders (number of individuals taken from the distribution with lower *α'*, individuals with higher probability of joining a movement). The bar colour is the proportion of observations for a given set of simulations, and thus ranges from 0 to 1, with lighter colours indicating higher values.

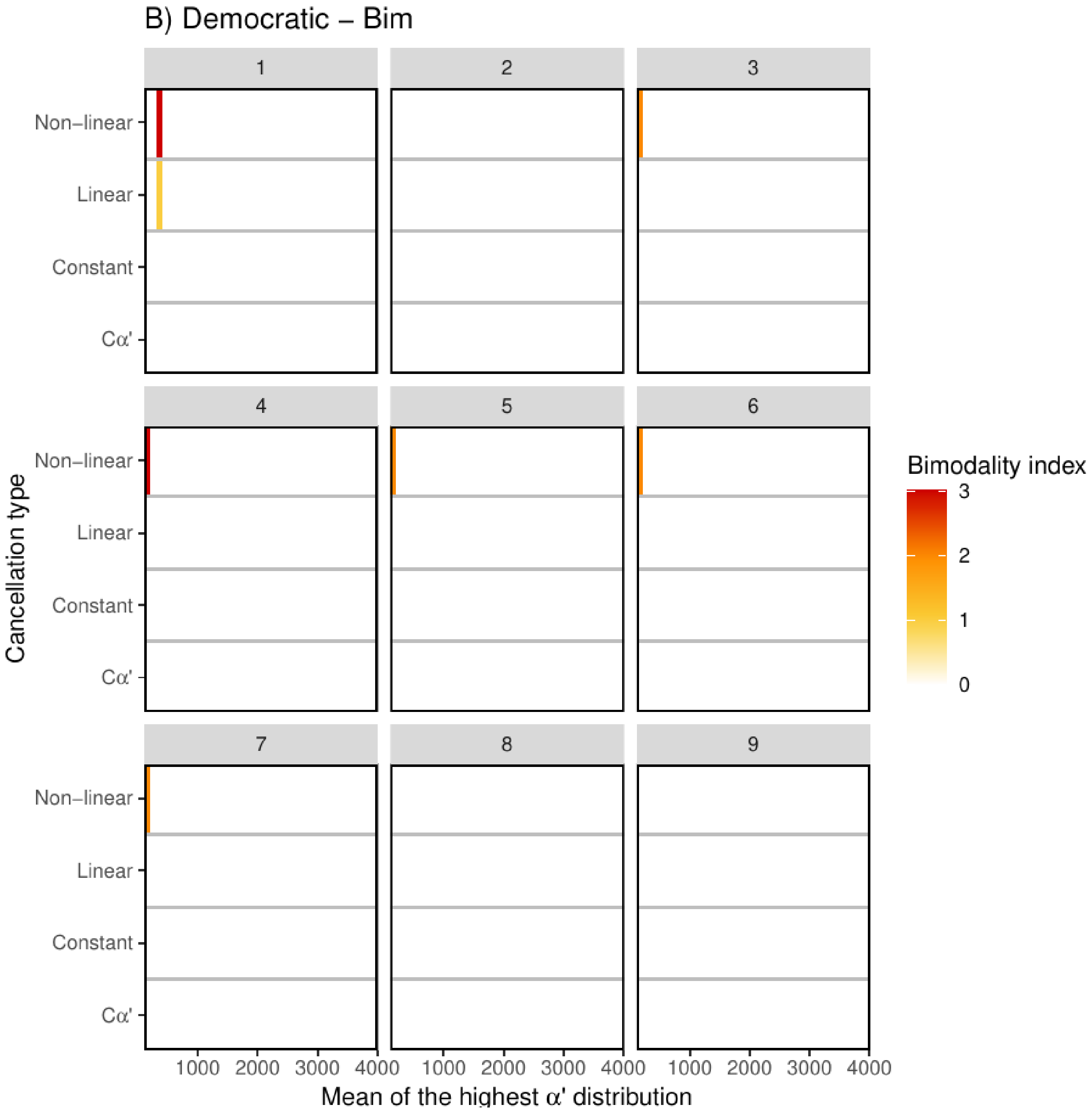

Figure S5. Scenarios where bimodality was produced under Bimodal condition for Democratic scenarios. y-axis indicates the cancellation type and the x-axis is the range of difference in α' values. The number on the top of each figure indicates the number of leaders. The first column of each graph corresponds to the control simulation without personality as $\alpha' = 160$ for all individuals. For the following columns the mean of the low α' distribution was fixed at 160, while the mean of the highest α' distribution is shown on the x-axis. The colour scale indicates the bimodality index (see methods for computation details); the higher the index, the higher the degree of bimodality.

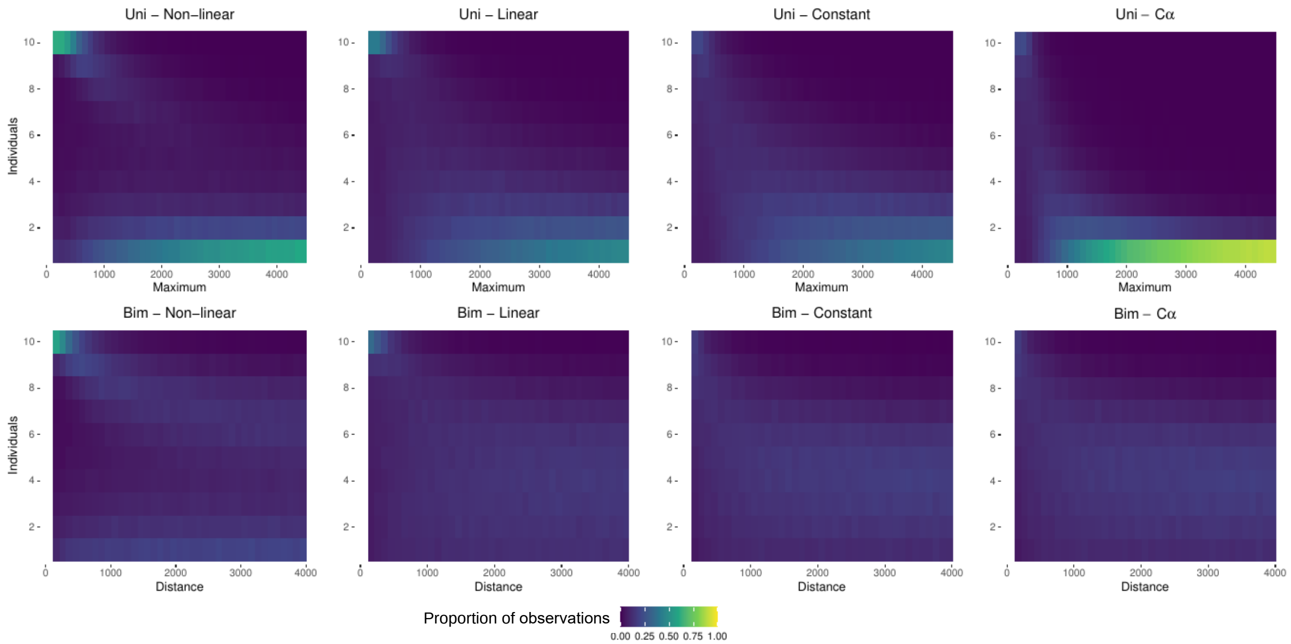

Figure S6. Results of the simulations for the Democratic scenario and Unimodal condition. Uni-non-linear corresponds to Unimodal condition with non-linear cancellation rate; Uni-linear is the Unimodal condition with linear cancellation rate; Uni-constant is the Unimodal condition with a constant cancellation rate; Uni-Cα is the Unimodal condition with cancellation rate proportional to α' (from the probability of joining). For comparison, we plot the Bimodal condition with a proportion of 5:5 leaders:followers. Bim-non-linear corresponds to Bimodal condition with non-linear cancellation rate; Bim-linear is the Bimodal condition with linear cancellation rate; Bim-constant is the Bimodal condition with a constant cancellation rate; Bim-Cα is the Bimodal condition with constant cancellation rate proportional to α' (from the probability of joining). The y-axis corresponds to the number of individuals following a movement. In the case of Unimodal condition, the x-axis is the maximum possible value of α' for a given simulation. In the case of Bimodal condition, the x-axis is the distance between both normal distributions used to create the bimodal distribution of α' (probability of joining a movement). The bar colour is the proportion of observations for a given set of simulations, and thus ranges from 0 to 1, with lighter colours indicating higher values.

Movie S2:

Legend of Movie S2: Summary of the results of the simulations for the Democratic scenario and Bimodal condition for different numbers of leaders. For Unimodal condition we used a uniform distribution of *α'* instead of a normal distribution. The results obtained with both distributions are qualitatively the same. From left to right, Non-linear corresponds to the Bimodal condition with non-linear cancellation rate; Linear is the Bimodal condition with linear cancellation rate; Constant is the Bimodal condition with a constant cancellation rate; Constant-alpha is the Bimodal condition with cancellation rate proportional to α' (probability of joining). The x-axis (N) corresponds to the number of individuals following a movement, the y-axis (Observation) is the proportion of observation of each number of individuals following a movement for a given set of simulations, and the z-axis (Mean) is the maximum value of the flat distribution of α' (probability of joining a movement). N Leaders on the top of each plot indicates the number of leaders (number of individuals taken from the distribution with lower α', individuals with higher probability of joining a movement). The bar colour, like the y-axis, is the proportion of observations for a given set of simulations, and thus ranges from 0 to 1.

2. Analytical Model

2.1. Constant probability of cancelling (P_C)

<u>*Despotic system*</u>

We explored the case where individuals differ on their P_J (and/or P_C), thus each individual k has its own ratio a_k:

$$a_k = \frac{P_{Jk}}{P_{Ck}} \quad (S1)$$

The global distribution of the number of followers is given by equation S2 (see description in main text):

$$\Theta^T(i,\infty) = \sum_{k=1}^{m} f_k \frac{N!}{(N-i)!} \frac{a_k^i}{\prod_{l=0}^{i} (a_k(N-l)+1)} ; i=0,\ldots,N \quad (S2)$$

In the case of groups in which one or more initiators is always followed by all potential followers (a has a very large value) and some initiators are never followed (a = 0), we obtain a bimodal distribution of the number of responders. However, the emergence of bimodality is not limited to this extreme case. The minimal condition to observe bimodality is that a fraction of the initiations has a ratio $P_J / P_C < 1$ and the other fraction has a $P_J / P_C > 1$. Numerical analyses limited to cases where we have 50% of individuals characterized as weakly efficient initiators or 'followers' ($a_1 > 1$) and 50% as efficient initiators or 'leaders' ($a_2 < 1$), show that the results can be divided in to two main type of outcomes. First, no bimodality is observed if we have a maximum of the distribution at 0 followers and a minimum at N (or visa versa). Second, we obtain a J or U-shape distribution with a maximum at 0 followers and at N followers and a minimum between 0 and N followers. Qualitatively, the bimodality is very robust but, not surprisingly, decreases with the difference between the two values of a_k. The bimodality index (BI) abruptly increases with the number of potential followers before to reach a plateau, and the plateau value depends on the difference between the two a values (Fig. S7).

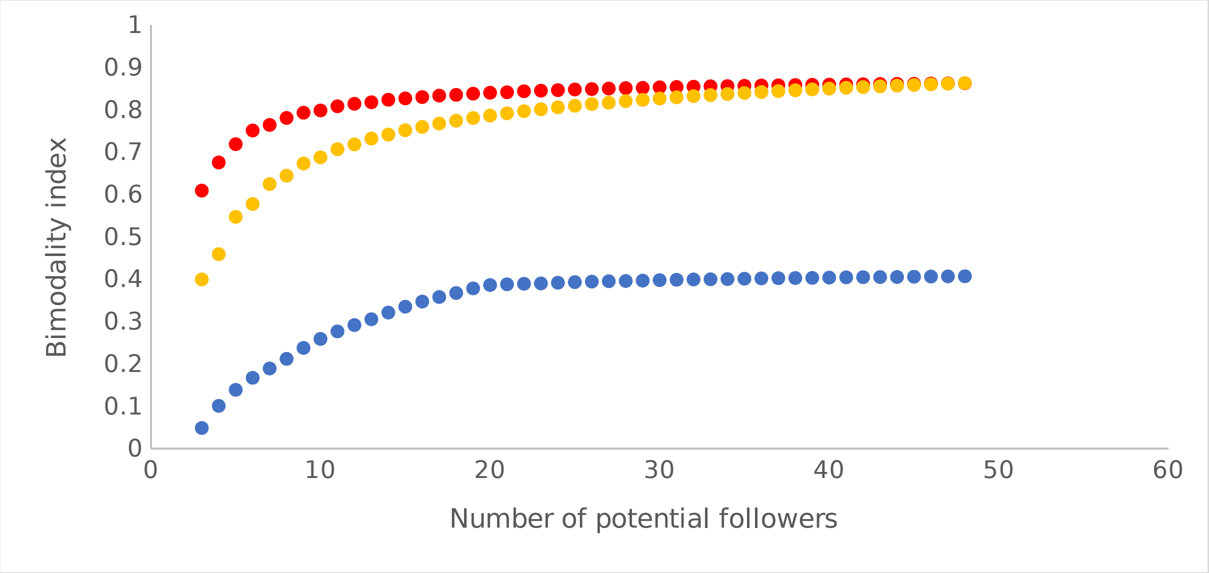

Figure S7: Bimodality index (BI) as a function of group size (N) and considering two types of individuals, the frequency (f) of which are $f_1 = f_2 = 0.5$. Colours indicate differences of a among two types of individuals: a_1 = 0.5, a_2 =1.5 (blue); a_1 = 0.1, a_2= 1.9 (orange); a_1 = 0.2, a_2 = 3.8 (red).

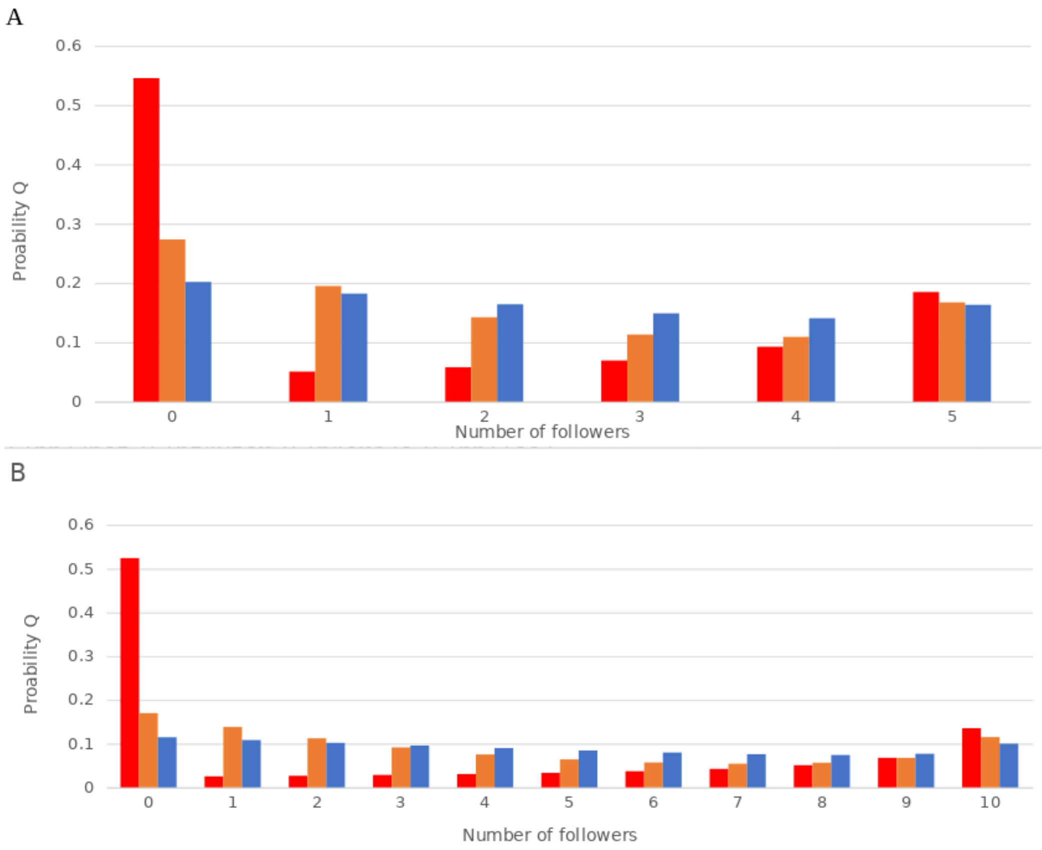

Figure S8: Probability of obtaining *i* followers with 5 (A) and 10 (B) potential followers. Results are obtained with 50% of individuals characterized as weakly efficient initiators or 'followers' (a_1) and 50% as efficient initiators or 'leaders' (a_2). The mean value of the two ratios a_i is equal to 1. Bars in blue correspond to initiators with a_1 = 0 .5 and a_2 = 1.5; orange corresponds to a_1 = 0.25 and a_2 = 1.75; red corresponds to a_1 = 0 and a_2 = 2.

Democratic system

We assume that different initiators have the same probability of cancelling P_C but that each follower is characterized by a probability P_{Ji} of following the initiator whatever his identity. In this case, we analysed a simplified situation where a group is characterized by two sub-groups of individuals, each sub-group with N_1 and N_2 individuals respectively, and with their own probability of following P_{J1} and P_{J2}. The probability that an initiator is followed by *i* (from sub-group 1) and *j* (from sub-group 2) individuals at time *t* is $\Theta(i, j, 1, t)$; while the probability that *i* and *j* individuals follow the initiator at time *t* and the latter abandons the movement is $\Theta(i, j, 0, t)$.These dynamics are described by the system of equations:

$$\frac{d\Theta(i,j,1,t)}{dt} = P_{J1}(N_1-(i-1))\Theta(i-1,j,1,t) + P_{J2}(N_2(j-1))\Theta(i,j-1,1,1,t)$$
$$-(P_{J1}(N_1-i)+P_{J2}(N_2-j)+P_C)\Theta(i,j,t); \quad i=1,\dots,N_1; \quad j=1,\dots,N_2 \qquad (S3,a)$$

$$\frac{d\Theta(0,j,1,t)}{dt} = P_{J2}(N_2-(j-1))\Theta(i,j-1,1,t) - (P_{J1}(N_1)+P_{J2}(N_2-j)+P_C)\Theta(i,j,t); \quad j=1,\dots,N \qquad (S3,b)$$

$$\frac{d\Theta(i,0,1,t)}{dt}=P_{J1}(N_1-(i-1))\Theta(i-1,j,1,t)-(P_{J1}(N_1-i)+P_C)\Theta(i,j,t);\quad i=1,\dots,N \quad (S3,c)$$

$$\frac{d\Theta(0,0,1,t)}{dt}=-(P_J(N_1)+P_{J1}(N_2)+P_C)\Theta(0,0,1,t) \quad (S3,d)$$

$$\frac{d\Theta(0,0,0,t)}{dt}=(-P_C)\Theta(0,0,1,t)(S3,e)$$

$$\frac{d\Theta(i,j,0,t)}{dt}=P_C\Theta(i,j,1,t);i=1,\dots,N_1,N_2(S3,f)$$

Numerical solutions show that the distribution of the number of followers is never bimodal. The probability of having *i* followers varies with the number of followers or has a maximum between both extremes of the distribution.

2.2. Probability of cancelling (P_C) decreases with the number of followers (Linear feedback)

In this case, a negative feedback is at work: the probability P_C decreases with the number of followers:

$$P_{Ci}=\frac{P_{C0}}{1+i} \quad (S4,a);\quad P_{Ci}=P_{Ci}=P_{C0}\left(1-\frac{i}{N}\right) \quad (S4,b)$$

Where P_{C0} is the intrinsic probability of cancelling and *i* is the number of followers. The results from equation S4,*a* are qualitatively identical to those obtained with equation S4,*b*. Results and figures reported here and in the main text have been obtained with equation S4,*a*.

As P_J / P_{C0} decreases, the ratio of $\Theta^T(N) / \Theta^T(0)$ decreases, but the BI increases and reaches a maximum at a critical value of P_J / P_{C0}, at which $\Theta^T(N) = \Theta^T(0)$, and the distribution of $\Theta(i)$ is more or less symmetrical (Fig. S9). This critical value decreases with $\approx N^{-0.6}$ (Fig. S10).

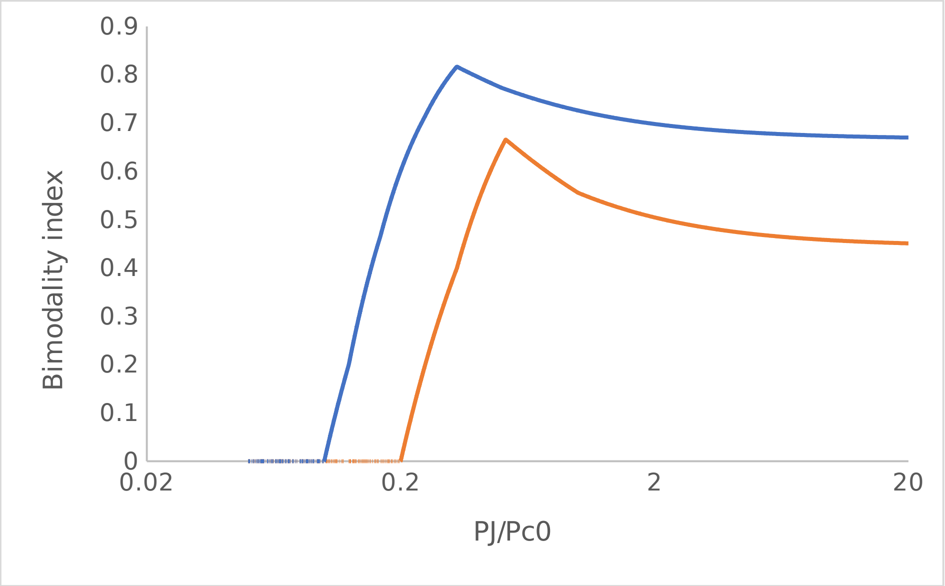

Figure S9: Bimodality index as a function of P_J / P_{C0} for 5 (orange) and 10 (blue) potential followers. When the bimodality index = 0, the distribution is monomodal.

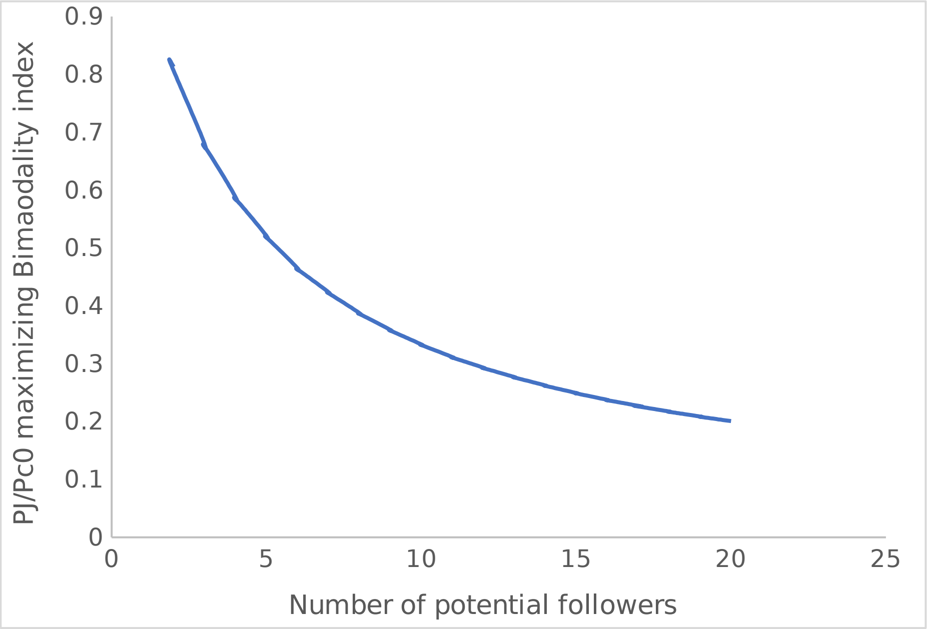

Figure S10: Ratio P_J / P_{C0} maximizing the bimodality index as a function of the number of potential followers.

While the analytical model predicts that the distribution of the number of followers can be bimodal under certain conditions, in practice this distribution is still very different from the pattern observed in empirical data. In such cases, non-linear feedback will give results in better agreement with natural patterns (see Petit et al., 2009).

References

Couzin, I.D., Krause, J., 2003. Self-Organization and Collective Behavior in Vertebrates. Adv. study Behav. 32, 1–75.

Gérard, C., Valenchon, M., Poulin, N., Petit, O., 2020. How does the expressiveness of leaders affect followership in domestic horses (*Equus ferus caballus*)? Anim. Cogn.

King, A.J., Cowlishaw, G., 2009. Leaders, followers and group decision-making. Commun. Integr. Biol. 2, 147–50.

Leca, J.B., Gunst, N., Thierry, B., Petit, O., 2003. Distributed leadership in semifree-ranging white-faced capuchin monkeys. Anim. Behav. 66, 1045–1052.

Petit, O., Gautrais, J., Leca, J.-B., Theraulaz, G., Deneubourg, J.-L., 2009. Collective decision-making in White-faced capuchin monkeys. Proc R. Soc B 276, 3495–3503.

Strandburg-Peshkin, A., Farine, D.R., Couzin, I.D., Crofoot, M.C., 2015. Shared decision-making drives collective movement in wild baboons. Science (80). 348, 1358–1361.

Sueur, C., Petit, O., 2008. Organization of Group Members at Departure Is Driven by Social Structure in Macaca. Int. J. Primatol. 29, 1085–1098.
