# Supplementary material for: Variation in personality can substitute for social feedback in coordinated animal movements
Source: Commun Biol. 2021 Apr 13;4:469. doi: 10.1038/s42003-021-01991-9 (PMC8044162; doi:10.1038/s42003-021-01991-9)
Supplement: Supplementary file 3 — Description of Additional Supplementary Files [file 42003_2021_1991_MOESM3_ESM.pdf]

Description of Additional Supplementary Files

File Name: Supplementary Movie S1

Description: Summary of the results of the simulations for the Despotic scenario and Bimodal condition for different numbers of leaders. For Unimodal condition we used a uniform distribution of α' instead of a normal distribution. The results obtained with both distributions are qualitatively the same. From left to right, Non-linear corresponds to condition with non-linear cancellation rate; Linear is the condition with linear cancellation rate; Constant is the condition with a constant cancellation rate; Constant-alpha is the conditions with the cancellation rate proportional to α' (probability of joining). The x-axis (N) corresponds to the number of individuals following a movement, the y-axis (Observation) is the proportion of observation of each number of individuals following a movement for a given set of simulations, and the z-axis (Mean) is the distance between both normal distributions used to create the bimodal distribution of α'(probability of joining a movement). N Leaders on the top of each plot indicates the number of leaders (number of individuals taken from the distribution with lower α', individuals with higher probability of joining a movement). The bar colour, like the y-axis, is the proportion of observations for a given set of simulations, and thus ranges from 0 to 1.

File Name: Supplementary Movie S2

Description: Summary of the results of the simulations for the Democratic scenario and Bimodal condition for different numbers of leaders. For Unimodal condition we used a uniform distribution of α' instead of a normal distribution. The results obtained with both distributions are qualitatively the same. From left to right, Non-linear corresponds to the Bimodal condition with non-linear cancellation rate; Linear is the Bimodal condition with linear cancellation rate; Constant is the Bimodal condition with a constant cancellation rate; Constant-alpha is the Bimodal condition with cancellation rate proportional to α' (probability of joining). The x-axis (N) corresponds to the number of individuals following a movement, the y-axis (Observation) is the proportion of observation of each number of individuals following a movement for a given set of simulations, and the z-axis (Mean) is the maximum value of the flat distribution of α'(probability of joining a movement). N Leaders on the top of each plot indicates the number of leaders (number of individuals taken from the distribution with lower α', individuals with higher probability of joining a movement). The bar colour, like the y-axis, is the proportion of observations for a given set of simulations, and thus ranges from 0 to 1.
